# Experimental Analysis and Modeling Study of Impedance Changes in Decellularized and Recellularized Peripheral Nerves

**DOI:** 10.3390/bioengineering13030344

**Published:** 2026-03-16

**Authors:** Marialourdes Ingrosso, Livio D’Alvia, Marianna Cosentino, Giorgia Nanni, Zaccaria Del Prete, Emanuele Rizzuto

**Affiliations:** 1 Unit of Hystology and Medical Embriology, Department of Human Anatomy, Histology, Forensic Medicine and Orthopedics, Sapienza University of Rome, 00161 Rome, Italy; maralourdes.ingrosso@uniroma1.it (M.I.); giorgia.nanni@uniroma1.it (G.N.); 2 Department of Well-Being, Health and Environmental Sustainability (BeSSA), Sapienza University of Rome, 02100 Rieti, Italy; livio.dalvia@uniroma1.it; 3 Department of Life Sciences, Health, and Health Professions, Link Campus University of Rome, 00165 Rome, Italy; ma.cosentino@unilink.it; 4 Department of Mechanical and Aereospace Engineering, Sapienza University of Rome, 00184 Rome, Italy; zaccaria.delprete@uniroma1.it

**Keywords:** peripheral nerve, tissue engineering, bioimpedance spectroscopy, multilayer equivalent circuit, nerve tissue regeneration

## Abstract

Peripheral nerve injuries pose a significant clinical challenge due to the limited self-repair capacity and the complexity of neural tissue architecture. Tissue engineering strategies applied to the peripheral nerve system aim to restore functional nerve constructs by combining scaffolds, cells, and biochemical cues to recreate the native microenvironment. This work aimed to propose the electrical conductivity as a functional readout of structural and biological remodeling in engineered peripheral nerve scaffolds, along with functional and molecular evaluations. To this end, bioimpedance measurements were combined with equivalent circuit modeling to track state-dependent changes across different levels of tissue organization. Murine sciatic nerves were decellularized and recellularized with neural populations to generate engineered constructs, and their electrical properties were assessed using broadband bioimpedance spectroscopy. Distinct impedance profiles were observed across control, decellularized, and recellularized samples, reflecting structural and functional changes associated with cell removal and repopulation. Furthermore, a multilayer series RC circuit model was implemented to accurately reproduce the measured spectra, enabling the extraction of layer-specific electrical parameters. Analysis of these parameters revealed that decellularization reduces compartmental resistances and increases inter-layer coupling, whereas recellularization restores outer-layer resistances and reduces coupling, consistent with functional tissue organization. Overall, the results demonstrate that bioimpedance provides a readout of the scaffold biological state and cellular integration, and that equivalent circuit modeling offers a quantitative framework to link structural remodeling to electrical function in engineered peripheral nerve tissues.

## 1. Introduction

**Figure 1:**
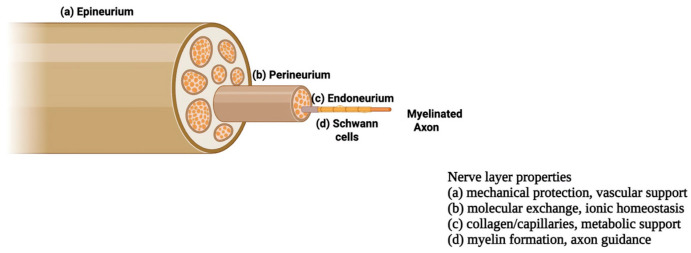
Schematic overview of the peripheral nerve with its three layers: epineurium, perineurium, and endoneurium, adapted from [14].

Regenerative medicine and tissue engineering (TE) aim to restore damaged tissues by integrating cells, scaffolds, and biochemical and physical cues to recreate the native physiological environment to generate functional three-dimensional constructs [1,2]. Within this framework, peripheral nerve tissue engineering has gained increasing attention, driven by the high clinical burden of nerve injuries and the intrinsic structural and functional complexity of nervous tissue. The peripheral nervous system (PNS) comprises spinal, cranial, and visceral nerves with their ganglia, consisting of neurons, glial, and stromal cells that relay signals between the CNS and body [3,4,5]. As shown in Figure 1, peripheral nerves exhibit a hierarchical organization, with the outer epineurium enclosing the entire nerve and providing mechanical protection and vascular support. Each fascicle is surrounded by the perineurium, which regulates molecular exchange and maintains ionic homeostasis. The innermost endoneurium layer wraps individual axons with collagen fibers and capillaries, supporting metabolic exchange. Within this compartment, Schwann cells encase axons, forming multilayered myelin around myelinated fibers, to accelerate conduction and, critically, to guide axonal regeneration after injury. Together with pericytes, they contribute to blood–nerve barrier integrity and tissue homeostasis [2,6,7,8,9,10,11,12,13].

Despite this organized architecture, peripheral nerve fibers are fragile and vulnerable to several types of damages [6,15], disrupting communication between the brain and muscles or organs. These damages occur in conditions such as peripheral nerve injury (PNI), causing severe and lasting impairments [7,16], and neurodegenerative diseases like amyotrophic lateral sclerosis (ALS) [17], where motor neurons and their distal axons degenerate, leading to muscle denervation. Nerve injury often leads to slow axonal regrowth (~1–3 mm/day), limited functional recovery, muscle atrophy, and chronic pain [6,8,18], with extensive tissue loss and prolonged denervation further increasing the risk of irreversible organ atrophy [6,16,19]. These challenges highlight the need for tissue-engineering strategies not only to bridge the nerve gaps but also to restore the neuro-muscular junction (NMJ) synapses and axon integrity in chronic conditions [20].

To address these challenges, peripheral nerve structural features must be considered not only for their mechanical and metabolic roles but also for their electrical behavior. The hierarchical organization of epineurium, perineurium, and endoneurium shapes how electrical currents propagate through the tissue. Horn et al. showed that the intrinsic electrical properties of PNS fiber layers control extracellular current flow, affecting activation thresholds, selectivity, and frequency-dependent responses during stimulation and recording [21], highlighting the central role of microstructure in nerve conductivity and underpinning the use of bioimpedance to assess nerve integrity and function. Bioimpedance reflects the frequency-dependent impedance from ionic conduction and membrane/interface polarization and serves as a label-free indicator of tissue state, with specific frequency ranges particularly sensitive to tissue composition, microstructure, and membrane integrity [22,23,24,25,26,27,28]. In the nervous system, neural electrical properties, including conductivity, are key for diagnostic and modulation approaches [29]. Although often approximated as homogeneous, neural tissue exhibits complex current pathways due to high-impedance membranes separating intra- and extracellular spaces and the dense, elongated organization of nerve fibers [30,31,32]. Beyond microstructure, neural tissue conductivity is influenced by experimental factors, including electrode type and geometry, contact quality, sample configuration and signal frequency [32]. Careful setup design is therefore fundamental to isolating the dielectric properties of the nerve from interface and parasitic effects. Indeed, the measurement configuration becomes as important as the tissue itself [22,26,33,34]. In simple two-electrode (bipolar) setups, a pair of electrodes is used for current injection and voltage sensing, so the measured spectrum is heavily influenced by electrode polarization and contact impedance at low frequencies and by parasitic capacitances and inductances at higher frequencies, often obscuring the intrinsic tissue response. In contrast, four-electrode (tetrapolar) configurations separate the current-carrying and sensing electrodes, strongly attenuating interface artefacts, enhancing sensitivity to the bulk and laminar properties of the nerve. An accurate measurement of the resistivity and permittivity of distinct tissue layers is therefore allowed. Reliable dielectric broadband measurements (≈10 Hz–100 MHz) are essential for accurate interpretation and comparison of data across the measured spectrum [21,25,26,33,34,35,36].

A detailed understanding of peripheral nerve microstructure and function is crucial for developing effective therapies, as peripheral nerve injuries (PNIs) present a major clinical challenge due to the limited self-repair capacity of the nervous system [37,38,39,40]. Current treatments combine surgical approaches, such as autografts and allogeneic grafts, with non-surgical strategies, including cellular therapies, biomaterials, and biochemical modulation, while tissue engineering using 3D scaffolds with cells and bioactive factors has emerged as a promising alternative [37,41,42,43,44,45,46,47,48,49,50].

The focus has shifted from simple nerve replacement to guided axonal regeneration and functional recovery, with engineered nerve guidance conduits (NGCs) that provide structural and biochemical cues mimicking native nerve architecture and showing efficacy comparable to autografts in long-gap injuries (>10–15 mm). Scaffold properties include electrical conductivity. among the possible strategies to fabricate scaffolds [19,51,52,53,54,55,56], decellularized native tissues represent an advanced platform to increase similarity to native tissue and are obtained by removing cellular components while preserving ECM architecture and bioactivity [41,43,57,58]. Decellularization relies on physical, chemical, or enzymatic methods [59,60,61,62,63,64,65,66,67,68,69] and can be further optimized through recellularization or integration of conductive pathways and electrical guidance cues [3,5,70,71,72,73,74].

In this context, the aim of this work was to investigate how controlled structural remodeling of peripheral nerve tissue, induced by decellularization and recellularization, modulates its electrical conduction properties. By comparing native, decellularized, and recellularized murine sciatic nerves, broadband bioimpedance measurements captured state-dependent changes in tissue organization, while equivalent circuit modeling served as an interpretative tool to link structural remodeling to functional electrical behavior in engineered peripheral nerve scaffolds.

## 2. Materials and Methods

### 2.1. Mouse Sciatic Nerve Harvesting and Sample Preparation

**Figure 2:**
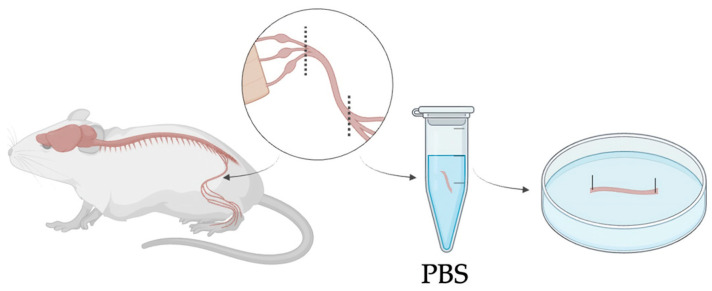
Schematic overview of mouse sciatic nerve harvesting, showing isolation from the hindlimb up to its trifurcation and positioning in a Petri dish with both ends fixed. Nerves were kept in PBS at room temperature to preserve hydration and integrity for a maximum of one hour before proceeding with the treatment.

All animal procedures adhered to ethical guidelines of the Declaration of Helsinki for experimentation and were approved by the Institutional Review Board of the animal facilities of the National Institute of Health-Italy. Mouse sciatic nerves were obtained from wild-type (WT) mice, aged 6–8 weeks. All mice were maintained in a temperature-controlled room (22 ± 1 °C) with a 12 h light–dark cycle and sacrificed via cervical dislocation immediately prior to the experiment. Following euthanasia, the skin was carefully removed from the hindlimb, and the sciatic nerve was exposed by making a deep incision above the knee. Using hooked forceps, the nerve was gently isolated from its origin to the beginning of its trifurcation into the tibial, sural, and peroneal branches (Figure 2). Excised nerves were directly transferred into Eppendorf tubes containing phosphate-buffered saline (PBS) to preserve tissue hydration and structural integrity and subsequently arranged in silicone-coated Petri dishes until the preparation of the experimental groups. Three different groups were identified: control nerves (CT), decellularized nerves (DEC), and recellularized nerves (REC).

To obtain the decellularized (DEC) samples, mouse sciatic nerves were processed using a method adapted from established procedures [75,76]. All the excised nerves were laid flat in silicone-coated Petri dishes and fixed at the edges to preserve the longitudinal orientation and to ensure uniform exposure to the decellularization solutions along their entire length. Tissues were rinsed in distilled water for 7 h at room temperature to promote osmotic cell lysis and facilitate decellularization while preventing cell preservation, incubated overnight in 3% Triton X-100 (Sigma-Aldrich, Merck KGaA, Darmstadt, Germany), and then treated with 4% sodium deoxycholate under constant agitation for 24 h. Through these steps, the acellular nerves (DEC) were obtained, with cellular components removed while preserving ECM integrity and native tissue architecture. The efficiency of decellularization and preservation of ECM was verified by immunofluorescence staining: Hoechst staining confirmed the absence of nuclei, and vimentin staining verified the structural integrity of the ECM. This structure represents a good candidate for a biological scaffold suitable for subsequent recellularization. The total time from nerve extraction to decellularization completion was 2 days.

**Figure 3:**
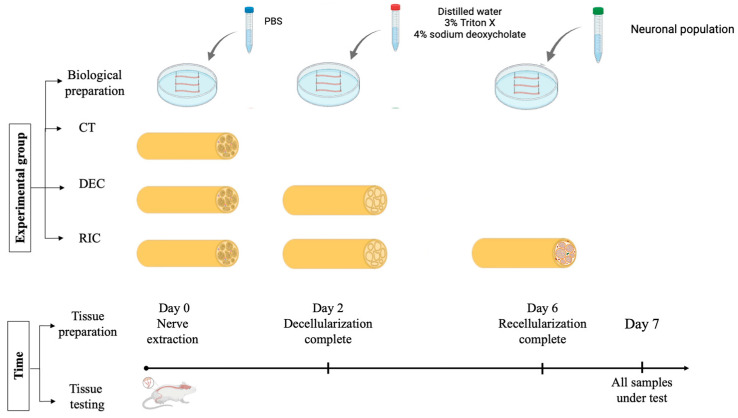
Experimental workflow for control (PBS-treated), decellularized and recellularized nerve scaffolds, and the experimental timeline leading to subsequent bioelectrical characterization. The total time from tissue excision to bioimpedance measurement was kept constant for all the groups.

Recellularized nerves (REC) were generated by seeding DEC scaffolds with primary motor neurons isolated from neonatal mouse spinal cords to ensure high cell viability and avoid the technical limitations of adult tissue [77,78,79]. Primary motor neurons were specifically used to recreate the motor component of the system, as our primary goal was to study direct innervation and efferent signal transmission. Neural tissue dissociation was performed using the Neural Tissue Dissociation Kit (Miltenyi Biotec, Bergisch Gladbach, Germany), according to the manufacturer’s instructions, combining enzymatic and mechanical steps to obtain a viable single-cell suspension. Cells were filtered, centrifuged, and resuspended in neural culture medium (DMEM supplemented with 1% fetal bovine serum (FBS), 1% L-glutamine, and 0.2% penicillin/streptomycin (Sigma-Aldrich, Merck KGaA, Darmstadt, Germany), 1% horse serum (Euroclone, Milan, Italy), and 2% B-27 supplement (Thermo Fisher Scientific, Waltham, MA, USA)) before seeding onto DEC scaffolds. As schematically shown in Figure 3, the decellularized nerves dedicated to recellularization were incubated with the neuronal cell suspension at a density of 250–500 cells/μL and maintained under standard culture conditions. Neuronal adhesion and early reinnervation of the nerve scaffold were observed within 4 days.

In accordance with the experimental timeline, the interval between nerve extraction and bioelectrical measurements was equal to the sum of the decellularization and recellularization periods and was the same for all three groups tested, with measurements performed 7 days post-extraction.

As a consequence, to obtain the control (CT) SHAM samples, the nerves selected for this group underwent the same procedure as DEC/REC nerves but were immersed in PBS, with medium changes matching those of the other groups, for the entire time required to reach day 7. Figure 3 summarizes the working group and the experimental timeline.

### 2.2. Experimental Setup and Impedance Measurements

**Figure 4:**
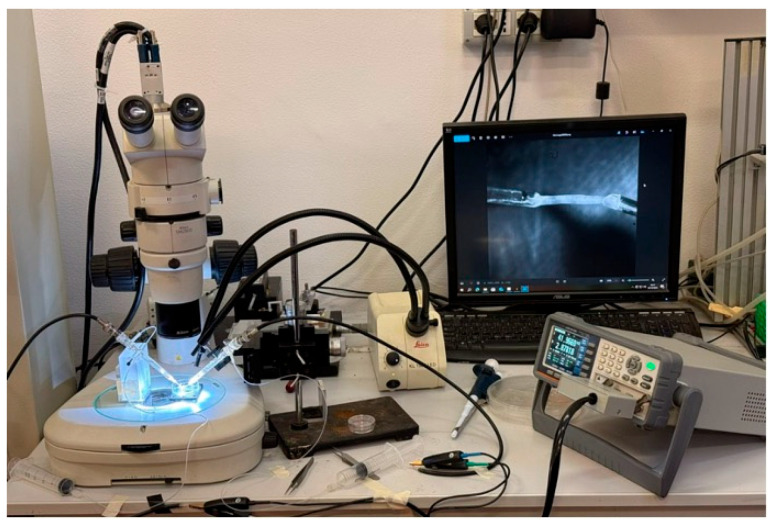
Experimental setup for electrical impedance measurements of sciatic nerve samples.

An experimental system for measuring the electrical impedance of sciatic nerve samples was developed and is schematically illustrated in Figure 4. Impedance measurements were performed using a RLC meter (RLC-6100, GW Instek, New Taipei City, Taiwan) in a two-electrode configuration with a four-wire (Kelvin) connection (via RS PRO LCR Meter Chip Test Fixture cables, RS Components Ltd., Corby, UK), which minimized lead resistance and cable parasitic contributions, improving measurement reliability. Given the particular shape, type, and dimensions of our specimens, we chose to use 2 single-suction silver electrodes (A-M Systems Inc., Sequim, WA, USA) to ensure stable, non-damaging attachment of the nerve ends during measurements. In this configuration, the tissue-electrode interface impedance was considered negligible compared to the measured tissue impedance within the selected frequency range, while the four-wire configuration electrically separates current-injection and voltage-sensing paths, dramatically reducing measurement artifacts. A stereomicroscope was integrated into the setup to ensure accurate alignment and positioning of the tissue, and the tissue’s images were acquired using a PC equipped with a CMOS camera (Basler aca-180 km, Basler AG, Ahrensburg, Germany), a frame grabber (NI PCIe-6251, National Instruments, Austin, TX, USA), and a NI LabVIEW 2018-based image acquisition software (National Instruments, Austin, TX, USA) for specimen length and diameter measurements, as previously described [80,81].

Impedance measurements were carried out by applying an alternating voltage, selected to electrically characterize the tissue without inducing stimulation or structural damage. The RLC meter was operated to record the impedance magnitude (|*Z*|). Measurements were acquired over a frequency range from 10 Hz to 80 kHz, a range typically explored for biological tissues’ characterization [27,82,83], with a step of 10 Hz. For each frequency point, three consecutive measurements were collected and averaged to reduce measurement variability. Impedance spectra obtained from these measurements were subsequently analyzed using an equivalent circuit modeling approach, as described in the following section.

### 2.3. Equivalent Circuit Modeling

To extract structural and functional information from global measurements and establish a robust link between tissue structure, biological remodeling, and electrical properties, an equivalent-circuit modeling approach was adopted. This strategy enabled the interpretation of the measured impedance spectrum in terms of physically meaningful circuit elements whose frequency-dependent behavior reflects the organization and interaction of sciatic nerve components. Model-based analysis enabled the decomposition of the measured global impedance into contributions from distinct conductive and dielectric regions, thereby allowing the modeling of internal tissue layers that are not directly accessible experimentally. Preliminary analyses were performed using the equivalent representations in series (Rs–Cs) and parallel (Rp–Cp) modes provided by the LCR meter. For each configuration, the corresponding impedance spectra were reconstructed and compared with the experimentally measured *|Z|*. A quantitative comparison using the Normalized Mean Squared Error (NMSE) showed lower values for the series-based model (0.0254) than for the parallel-based model (0.1005). This comparison indicated that series-based representations more accurately reproduced the nerve’s frequency-dependent behavior.

**Figure 5:**
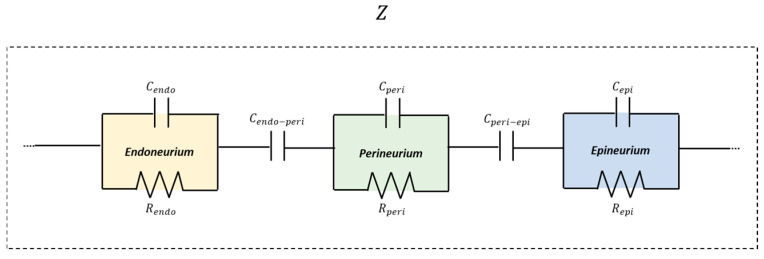
Schematic representation of the multilayer equivalent circuit model of nerve tissue representative layer compartments.

According to this preliminary result, the peripheral nerve was modeled as a multilayer structure composed of three electrically distinct compartments, to resemble endoneurium, perineurium, and epineurium, arranged in series. Each compartment was represented by a parallel resistance–capacitance (RC) element. Within this framework, resistive elements account for ionic conduction through extracellular and intracellular pathways, while capacitive elements represent charge accumulation and polarization effects associated with membranes, laminar interfaces, and dielectric barriers. This architecture captured the presence of multiple characteristic time constants observed experimentally, which cannot be reproduced by simpler single-RC or purely resistive models. To further improve the correspondence between the model and the real tissue, and to increase fitting accuracy, two capacitive coupling elements were introduced between adjacent RC layers ($C_{endo-peri}$ and $C_{peri-epi}$). These coupling capacitances account for the electrostatic interaction between neighboring compartments arising from their close spatial proximity and the presence of dielectric structures. Modeling through capacitors adequately reproduces the restriction in direct current flow caused by nerve layers. The inclusion of capacitive coupling introduced additional characteristic time constants into the model, enabling it to reproduce the multiple slope changes observed in the experimental *|Z|* curves. Figure 5 shows a schematic representation of the adopted circuit model.

Within this modeling framework, parameter estimation was performed by fitting the model impedance to the measured *Z* values using nonlinear least-squares optimization (MATLAB-2021 “*lsqcurvefit*”). The fitted output parameters provided effective electrical descriptors whose variations across conditions reflected nerve reorganization following decellularization and recellularization. This modeling framework linked impedance features to structural remodeling, supporting bioimpedance as a label-free probe of engineered peripheral nerve tissue. The proposed equivalent circuit model was further extended to investigate how neuronal repopulation of the decellularized nerve scaffold affects tissue’s impedance. Since the ECM architecture and the multilayer organization of the nerve are preserved after decellularization, neuronal integration was assumed to be spatially non-uniform rather than evenly distributed across all layers. Based on the biological characteristics of the scaffold (i.e., the ECM) and the cell-seeding procedure, it was hypothesized that neuronal adhesion predominantly occurs at the outermost layer. To test this hypothesis, different fitting strategies were applied. First, a fully unconstrained model was considered, in which all resistive and capacitive parameters, including inter-layer coupling capacitances, were allowed to vary freely, representing an integration of the neuronal population at the entire tissue levels. Subsequently, 3 constrained models were implemented in which the parameters’ variation was limited to a single layer and their associated coupling capacitance at each time step, while all the other parameters were kept fixed at the initial decellularized condition. Separate fits were therefore performed by allowing variations in the endoneurial, perineurial, or epineurial compartments, respectively. Only selected subsets were allowed to vary:

- Endoneurial RC and its adjacent coupling capacitance ($R_{endo}$,$C_{endo}$,$C_{endo-peri})$
- Perineurial RC and adjacent coupling capacitances ($R_{peri}{,C}_{peri}$,$C_{endo-epi}$,$C_{peri-epi}$)
- Epineurial RC and its adjacent coupling capacitance ($R_{epi}$,$C_{epi}$,$C_{peri-epi}$).

The initial values of the parameters required by the *lsqcurvefit* function for model fitting were obtained starting from literature-reported electrical properties of peripheral nerves and adapted based on geometric and physical considerations [21,84,85]. Capacitances and resistances for each nerve layer were calculated using classical relations expressed in Equations (1) and (2):

(1) $$C_{layer}=\varepsilon_{0}\varepsilon_{r}\frac{A}{d}$$

(2) $$R_{layer}=\rho \frac{l}{A}$$

where ρ is the resistivity, $\varepsilon_{0}$ the vacuum permittivity, $\varepsilon_{r}$ the relative layer permittivity, *l* the conduction length, A the area perpendicular to the flow of the electrical phenomenon being considered, and *d* the thickness of each nerve’s layer. Based on length ad cross sectional area measurement performed on each tested specimen, a representative nerve length of 5 mm and external cross-sectional area of 0.5 mm^2^ were assumed for the outer layer (epineurium). The dimensions for the inner concentric layers were then obtained using anatomical scaling ratios derived from literature measurements of epineurium, perineurium [21,84] and endoneurium [84,85]. The same authors provided electrical properties (ρ and $\varepsilon_{r}$) for each layer. From these values, layer resistances and capacitances were calculated according to Equations (1) and (2) and are shown in Table 1. These parameters served as physiologically informed initial values for model fitting (Table 2). and were subsequently refined through iterative fitting to match the experimental data, remaining within the same order of magnitude as the calculated estimates [29,86,87,88]. Once the fitting parameters were determined, the upper and lower bounds were defined around these values to constrain the fitting within a plausible physiological range, ensuring the model could explore parameter variations without diverging into unrealistic values. Inter-laminar coupling capacitances were defined as fractions of neighboring layer self-capacitances, remaining smaller than the minimum of the adjacent values, consistent with established models of layered biological tissues [89,90].

Statistical analysis was performed using GraphPad Prism 10 software. Differences in the values of impedance magnitude were evaluated using a two-way ANOVA using the *nerve type* and frequency (frequency was considered a repeated measure factor). Post hoc multiple comparisons tests were employed to assess pairwise differences using Tukey’s multiple comparisons test to correct for multiple testing. Frequency-dependent effects within each experimental group were assessed separately using repeated-measures one-way ANOVA, treating frequency as a within-subject factor, followed by post hoc multiple comparisons to identify pairwise significant differences. Data are presented as mean ± standard deviation (SD), and statistical significance was accepted at *p* < 0.05.

## 3. Results

### 3.1. Impedance Spectra of Control, Decellularized, and Recellularized Nerves

**Figure 6:**
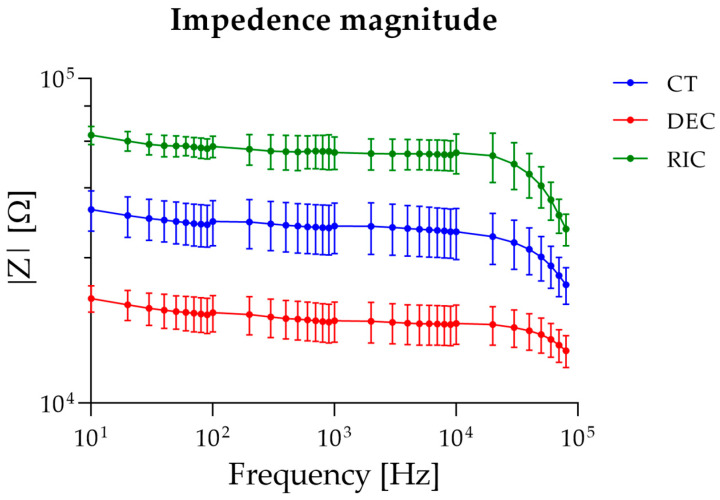
Bioimpedance spectra of sciatic nerve samples.

Figure 6 shows the frequency dependence of the electrical impedance magnitude *|Z|* measured in sciatic nerve samples under control, decellularized, and recellularized conditions over the frequency range 10 Hz–80 kHz. In all experimental groups, *|Z|* decreased with increasing frequency, exhibiting the behavior typical of biological tissues. Statistical analysis revealed a highly significant effect of the nerve type (*p* < 0.0001), accounting for approximately 85% of the total variance. This result indicated that the tissue’s biological state is the primary determinant of the impedance response. A significant effect of frequency was also observed (*p* < 0.0001), consistent with the dielectric behavior of biological tissues, together with a significant nerve type × frequency interaction (*p* < 0.0001), confirming that the differences between groups depend on the analyzed frequency regime. Post hoc analyses confirmed statistically significant differences between all group pairs across the entire frequency range considered (*p* < 0.001), demonstrating the robustness of the experimentally observed differences. These differences were reported throughout the entire frequency range and were more pronounced at low frequencies, where interfacial polarization and cellular compartmentalization effects dominate.

To further assess the effect of frequency within each experimental condition, one-way ANOVA was applied separately to control, decellularized, and recellularized sciatic nerve groups. For all the tested groups, one-way ANOVA revealed a significant effect of frequency on impedance magnitude. In the control group, post hoc multiple comparisons indicated that impedance values were statistically similar at low frequencies (10–20 Hz), began to diverge at intermediate frequencies (30–5000 Hz), and showed distinct decreases at higher frequencies (>50 kHz), consistent with progressive frequency-dependent impedance reduction. In decellularized nerves, post hoc analysis showed comparable impedance at low frequencies (10–30 Hz), a largely uniform plateau across intermediate frequencies (40 Hz–50 kHz), and statistically lower impedance at the highest frequencies (60–80 kHz). Finally, for recellularized nerves, post hoc comparisons revealed similar impedance at the lowest frequencies (10–30 Hz), followed by moderate differences across intermediate frequencies (40 Hz–10 kHz), and marked decreases at high frequencies (>20 kHz), with the most substantial changes observed above 40 kHz.

Since |Z| depends on both the length (*l*) and cross-sectional area (S) of the tissue under test, we subsequently proceeded by normalizing the impedance values with respect to geometric dimensions. Indeed, the length of the tissue subjected to the flowing current during experimental testing depends on the extraction procedure and its insertion into the suction electrodes. Moreover, decellularization and recellularization procedures may affect the tissue cross-sectional area.

**Figure 7:**
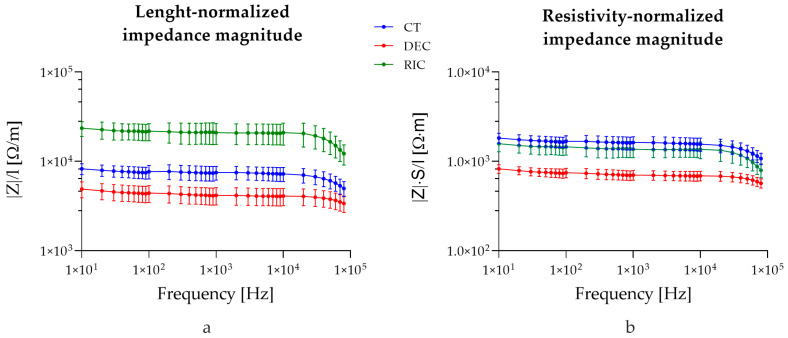
Impedance values normalized on tissue length (**a**) and tissue length and cross-sectional area (**b**).

Figure 7a shows the impedance normalized by sample length (Z/l) across control (CT), decellularized (DEC), and recellularized (RIC) sciatic nerve samples, assuming the system as a bioelectric line type. Two-way ANOVA revealed significant effects of nerve type (*p* < 0.0001), frequency (*p* < 0.0001), and their interaction (*p* < 0.0001). Post hoc comparisons test confirmed that the differences observed between all experimental groups remained statistically significant across the analyzed frequency range. These findings indicated that normalization by length does not alter the overall hierarchy observed in the non-normalized impedance spectra, and that variations in sample length contribute minimally to the observed impedance changes.

Figure 7b shows the impedance normalized for both cross-sectional area and length (|Z| × S/l). The normalization of the measured impedance by geometrical factors (S/L) yields an effective complex resistivity $\rho^{*}(\omega )$, representing a frequency-dependent electro structural property of the nerve, emerging from the interaction of its multilayer architecture and inter-compartment capacitive coupling (Equation (2)). Two-way ANOVA revealed significant effects of group (*p* < 0.0001), frequency (*p* < 0.0001), and their interaction (*p* < 0.0001). Post hoc comparisons showed that decellularized tissue differed significantly from control at all frequencies (*p* < 0.0001). Moreover, recellularized samples were significantly higher than decellularized samples from 10 Hz up to ~10 kHz, while differences at higher frequencies became not significant. No significant differences were observed between control and recellularized samples across most of the analyzed spectrum, with only the highest frequency (80 kHz) showing a minor significant difference (*p* = 0.0397). Interestingly, no significant difference was reported between the recellularized and control groups, confirming that the intrinsic conductivity of the recellularized tissue was comparable.

### 3.2. Equivalent Circuit Model Fitting and Layer-Specific Parameter Changes

**Figure 8:**
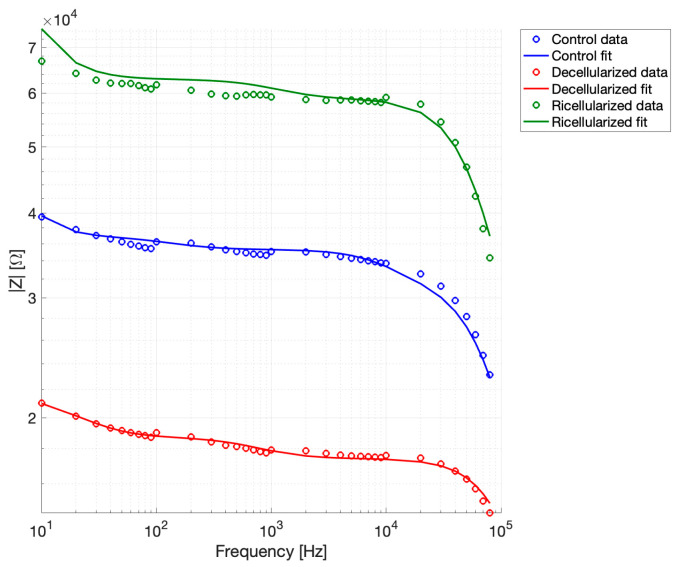
Experimental impedance spectra and multilayer RC model fits.

The experimental impedance spectra were fitted using the multilayer series RC model with inter-layer capacitive coupling as described above. Figure 8 shows the average values of the measured |Z| and the corresponding fitted curves for all the experimental groups.

Goodness-of-fit metrics were computed to assess agreement between experimental data and model predictions, and the complete results for each dataset are reported in Table 3. Linear, relative, and logarithmic residuals were considered to provide a comprehensive evaluation of model performance. These metrics help to understand how closely the model follows the experimental data, with smaller RMSE and residuals indicating better agreement, and high R^2^ values showing that the model captures most of the trends observed in the measurements. As reported, the fitting accuracy was very high across all experimental groups, with RMSE values always below 5% and R^2^ > 0.9. Low residuals in both linear and logarithmic scales confirm that the model accurately reproduces both overall trends and finer variations in the impedance across the measured frequency range. Residuals remained low in both linear and logarithmic scales, indicating good agreement between model predictions and experimental data over the entire frequency range. The reduced chi-square values further suggested that the model adequately reproduces the frequency-dependent impedance behavior of the tissue. Overall, these results confirmed that the equivalent circuit model provides a robust and accurate description of the electrical properties of peripheral nerve tissue under different biological states.

Table 4 summarizes the resistive and capacitive elements of the endoneurial, perineurial, and epineurial compartments, as well as the inter-layer coupling capacitances, for control, decellularized, and recellularized samples.

**Figure 9:**
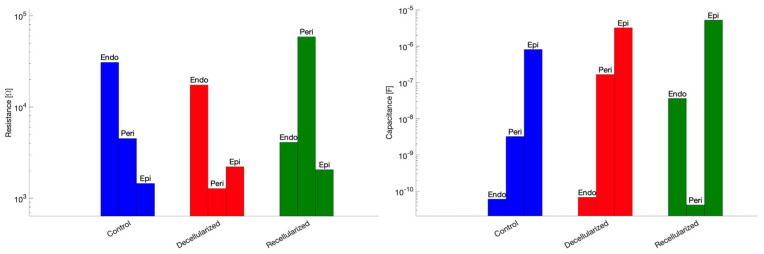
Fitted resistance (**left**) and capacitance (**right**) parameters across datasets.

In addition, a graphical comparison of the fitted resistance and capacitance parameters across datasets is shown in Figure 9, allowing a clearer visualization of parameter variations between experimental conditions.

Interestingly, decellularization reduced resistances in all compartments, particularly in the endoneurial and perineurial layers. Capacitive elements vary due to fluid redistribution changes in the inter-layer coupling. Recellularization restored resistances in the endoneurial and perineurial layers and partially restored compartment-specific capacitances. Coupling capacitances decrease in recellularized samples.

**Figure 10:**
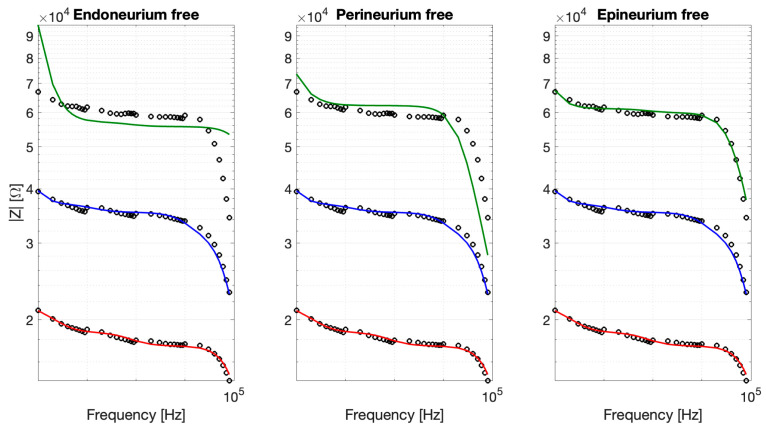
Constrained fits obtained by allowing one anatomical layer at a time to vary while keeping the remaining parameters fixed (CT, blue; DEC, red; REC, green).

To explore the layer-specific contribution of neuronal repopulation to the impedance changes observed after recellularization, constrained fitting strategies were evaluated. Figure 10 summarizes the results for the fitting parameters associated with endonerium, perinerium, and epinerium free, respectively. The analysis revealed distinct layer-specific effects of neuronal integration on the electrical impedance of the decellularized nerve scaffold. Allowing only the epineurial parameters to vary yielded the best agreement with the experimental data (RMSE 2.54%, R^2^ = 0.97), accurately reproducing the increased impedance observed in recellularized samples. In contrast, varying the perineurial parameters resulted in reduced performance (RMSE 9.20%, R^2^ = 0.59), while freeing the endoneurial parameters failed to capture the observed impedance changes (RMSE 15.65%, R^2^ = −0.17). Overall, these results confirmed our hypothesis that neuronal adhesion predominantly occurs at the outermost layer.

**Figure 11:**
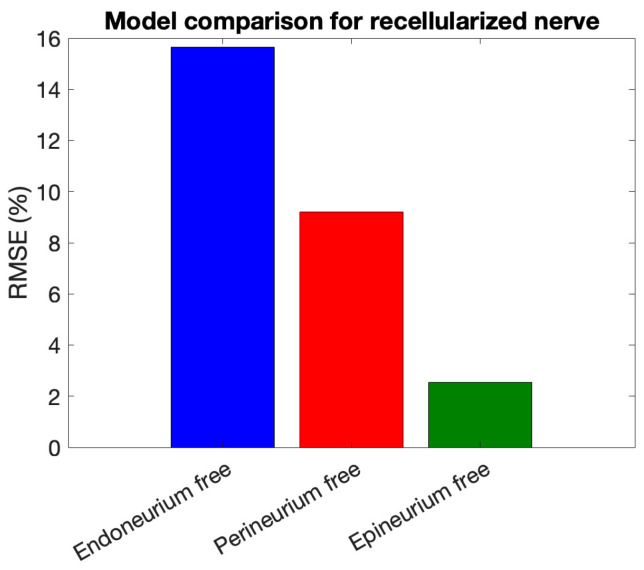
RMSE (%) of layer-specific models for the recellularized nerve.

To provide a quantitative comparison of model performance, Figure 11 shows a bar plot of the RMSE (%) for the three layer-specific models tested on the recellularized nerve dataset, highlighting the layer mainly contributing to the impedance fit.

Finally, it has to be remarked that the proposed modeling framework incorporates group-specific geometric parameters (length and cross-sectional area), allowing discrimination between structural size effects and intrinsic electrical property changes.

## 4. Discussion

The experimental data consistently showed a clear impedance hierarchy, with ${\left|Z\right|}_{RIC}>{\left|Z\right|}_{CT}>{\left|Z\right|}_{DEC}$ across the entire frequency range, particularly at low frequencies where interfacial polarization and tissue compartmentalization dominate. Decellularization significantly reduced impedance, particularly at low frequencies where cellular membranes contribute most. Biologically, this effect arose from the removal of cells, plasma membranes, and intercellular junctions. Electrically, the removal of insulating structures simplifies the tissue microstructure, reduces charge storage, and promotes direct ionic conduction, thereby increasing effective conductivity and lowering global impedance. This indicated a less pronounced frequency-dependent decrease compared with control nerves. These findings also confirmed the functional effectiveness of the decellularization process. On the other hand, recellularized samples exhibited significantly higher impedance than both control and decellularized tissues across the entire frequency range, with the largest differences at low and intermediate frequencies, where polarization and compartmentalization dominate. This increase reflected the reintroduction of cellular membranes and the definition of intra- and extracellular domains, enhancing charge storage and interfacial polarization, and restoring a more physiologically structured electrical response. These differences remained consistent after normalization for sample length, confirming that the hierarchy reflects intrinsic electrical property changes rather than geometric variability. When further normalizing for cross-sectional area, recellularized tissue exhibited resistivity values overlapping with control nerves and clearly distinct from decellularized samples. Taken together, these results confirmed the high impact of the recellularization process on tissue impedance, with this impact due to recovery in tissue resistivity and, in part, to geometrical changes in the tissue (mainly a reduction in the cross-sectional area).

Accordingly, decellularized tissue appeared electrically simplified, showing fewer significant differences across frequencies. In contrast, recellularization restored a frequency-dependent impedance hierarchy, with significantly higher values at low-to-intermediate frequencies, consistent with functional tissue remodeling. This pattern supports bioimpedance as a sensitive marker of cellular integration, reflecting the partial recovery of compartmentalized electrical properties within the tissue.

All these findings agreed with the observed frequency-dependent variation in impedance magnitude, as revealed by the one-way ANOVA and multiple comparisons performed within each experimental group. In control nerves, distinct frequency clusters reflected the presence of electrically active cellular compartments, while the reduced number of significant differences in decellularized tissue indicated a simpler electrical structure due to cell removal. Recellularized nerves showed restored, more complex frequency segmentation, consistent with renewed cellular architecture. This further confirmed that impedance measurements sensitively capture the frequency-specific electrical properties linked to tissue microstructure.

The experimental findings are complemented by equivalent circuit modeling, which provided a mechanistic interpretation by decomposing the global impedance into compartment-specific contributions. The final model outputs represented effective, frequency-dependent properties at the measurement scale, based on the lumped-element descriptions of neural tissue. The obtained parameters clarified how decellularization and recellularization reshape the nerve’s electrical organization. Decellularization reduced resistances across all compartments, particularly the endoneurial and perineurial layers, reflecting the removal of cellular membranes and the simplification of microstructural barriers. Capacitive variations reflected fluid redistribution, while increased inter-layer coupling indicated diminished compartmentalization. In contrast, recellularization restored compartmental resistances and partially recovered layer-specific capacitances, consistent with the reintroduction of cellular membranes and the establishment of defined intra- and extracellular domains. The observed reduction in coupling capacitances further suggested improved electrical isolation between layers. This elevated impedance indicates a functional, compartmentalized tissue architecture capable of supporting controlled bioelectrical signal propagation. Overall, the model revealed that recellularized tissue recovered a structured, compartmentalized electrical architecture capable of supporting controlled bioelectrical signal propagation. These findings underscored the physiological relevance of the equivalent circuit framework and reinforced the value of bioimpedance as a quantitative tool for monitoring tissue remodeling.

By isolating layer-specific contributions, the model directly mapped impedance changes to cellular localization within specific layers, providing a clearer and more interpretable description than fully unconstrained fits. While the fully free fit captures all impedance changes, it does not reveal which layers drive these changes. Of note, preserving the epineurium during the experiments was crucial to maintain structural integrity and mimic the native nerve environment. Future investigations may involve epineurium removal to specifically address its contribution to global impedance [91]. In contrast, freeing only the epineurial parameters reproduces the experimental data with higher precision and directly links the impedance increase to the outermost layer. This comparison highlighted the predominance of the epineurium in mediating electrical changes during neuronal recellularization, with negligible contributions from the endoneurium and minor effects from inter-layer coupling.

Overall, these results demonstrate that constrained, layer-specific modeling provides a biologically informative mapping of cellular integration onto electrical properties, offering insights that fully free fits alone cannot reveal. This approach highlights the sensitivity of bioimpedance as a tool for monitoring neuronal recellularization. While in vivo translation may involve additional factors such as immune response, vascularization, and microenvironmental complexity, our in vitro model provides controlled mechanistic insights into motor neuron integration and scaffold function. Future in vivo studies will be attempted to assess functional regeneration.

## 5. Conclusions

Electrical impedance spectroscopy across control, decellularized, and recellularized nerve samples revealed distinct frequency-dependent responses that reflect the tissue’s structural and cellular organization. Decellularization reduced impedance values, consistent with the removal of cellular membranes and simplification of microstructural barriers, whereas recellularization increased impedance, particularly in the outer layers, indicating effective restoration of compartmentalized, electrically functional tissue. Fitting the experimental data to a multilayer RC model with inter-layer capacitive coupling enabled the interpretation of these changes in physiologically meaningful terms. Fully free fits reproduced the overall impedance spectra, confirming that the engineered scaffolds acquire a globally coherent electrical organization. Layer-specific constrained analyses further identified the epineurial layer as the main contributor to the observed impedance increase. Overall, these findings demonstrate that bioimpedance provides a sensitive, non-destructive readout of scaffold biological state, and that combining experimental measurements with layer-specific modeling allows quantitative mapping of cellular integration onto electrical properties. This approach highlights the potential of impedance spectroscopy to monitor functional tissue remodeling and supports the design of engineered peripheral nerve constructs with physiologically relevant electrical behavior.

## Figures and Tables

**Table 1:** Layer dimensions and electrical measures.

Layer	Diameter (mm)	Thickness (μm)	Length (m)	Resistivity (Ω·m)	Relative Permittivity $\left(\varepsilon_{r}\right)$
Endoneurium	0.18	1.60	0.005	1 [81,82]	80 [81,82]
Perineurium	0.30	2.73	0.005	3751.3 [19,81]	2018 [19,81]
Epineurium	0.504	36.4	0.005	55 [19,81]	$4.97\;\times \;10^{6}$ [19,81]

**Table 2:** Initial model parameters used for fitting.

Layer	R (Ω)	C (F)	C Coupling (F)
Endoneurium	3.0 × 10^3^	1.15 × 10^−11^	$C_{endo-peri}\;$= 1.0 × 10^−11^
Perineurium	$1.0\;\times {\;10}^{8}$	$1.0\;\times \;10^{-11}$	
Epineurium	1.37 × 10^3^	3.0 × 10^−7^	$C_{endo-epi}$ = 5.0 × 10^−11^

**Table 3:** Goodness-of-fit metrics for control, decellularized, and recellularized samples.

Dataset	SSR_rel	RMSE (%)	SSR_log	RMSE_log	χ^2^_red	R^2^
CT	0.011	1.74	0.0020	0.0076	7.46 × 10^−5^	0.9740
DEC	0.0039	1.05	7.22 × 10^−4^	0.0045	2.67 × 10^−5^	0.9814
REC	0.045	3.59	0.0080	0.0151	2.96 × 10^−4^	0.9071

**Table 4:** Fitted resistive and capacitive parameters of nerve layers and inter-layer couplings.

Parameter	CT	DEC	REC
$R_{endo}$ (Ω)	3.08 × 10^4^	1.74 × 10^4^	4.09 × 10^3^
$C_{endo}$ (F)	6.07 × 10^−11^	6.79 × 10^−11^	3.58 × 10^−8^
$R_{peri}$ (Ω)	4.52 × 10^3^	1.28 × 10^3^	5.87 × 10^4^
$C_{peri}$ (F)	3.22 × 10^−9^	1.66 × 10^−7^	4.19 × 10^−11^
$R_{epi}$ (Ω)	1.45 × 10^3^	2.21 × 10^3^	2.05 × 10^3^
$C_{epi}$ (F)	8.16 × 10^−7^	3.19 × 10^−6^	5.23 × 10^−6^
$C_{endo-peri}$ (F)	2.14 × 10^−6^	8.83 × 10^−6^	8.47 × 10^−7^
$C_{peri-epi}$ (F)	2.23 × 10^−6^	9.19 × 10^−6^	8.79 × 10^−7^

## Data Availability

Data are available upon request to the authors.
